# Supplementary material for: Twelve-Week Safety and Potential Lipid Control Efficacy of Coffee Cherry Pulp Juice Concentrate in Healthy Volunteers
Source: Nutrients. 2023 Mar 25;15(7):1602. doi: 10.3390/nu15071602 (PMC10097379; doi:10.3390/nu15071602)
Supplement: Supplementary file 1 [file nutrients-15-01602-s001.zip › nutrients-2283606-supplementary.pdf]

Supplemental Material

Sample size calculation

The sample size of the present study was calculated by using G-power V.3.1.9.4 (Heinrich-Heine-Universität Düsseldorf, Düsseldorf, Germany) [19]. The theoretical large effect size value of 0.8 for the comparison of two independent means (the unpaired t-test) was used. With a power of 0.8 and a significance level of 0.05, the calculation of sample size for two-tailed yielded the result of at least 26 people per group as shown below. Assuming a dropout rate of 15%, the sample size was set at thirty people (n = 30) in each group, and the total sample size was 60.

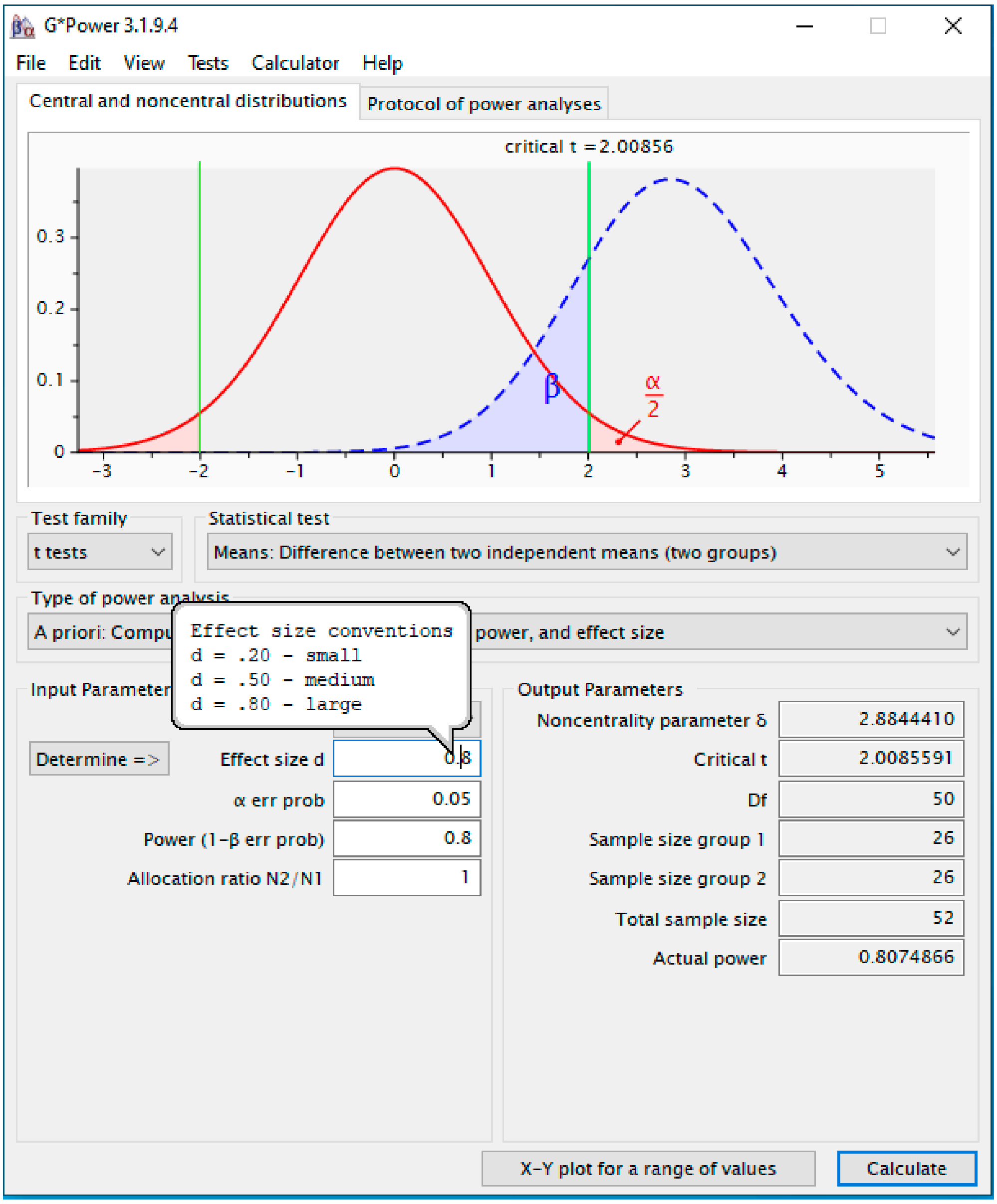

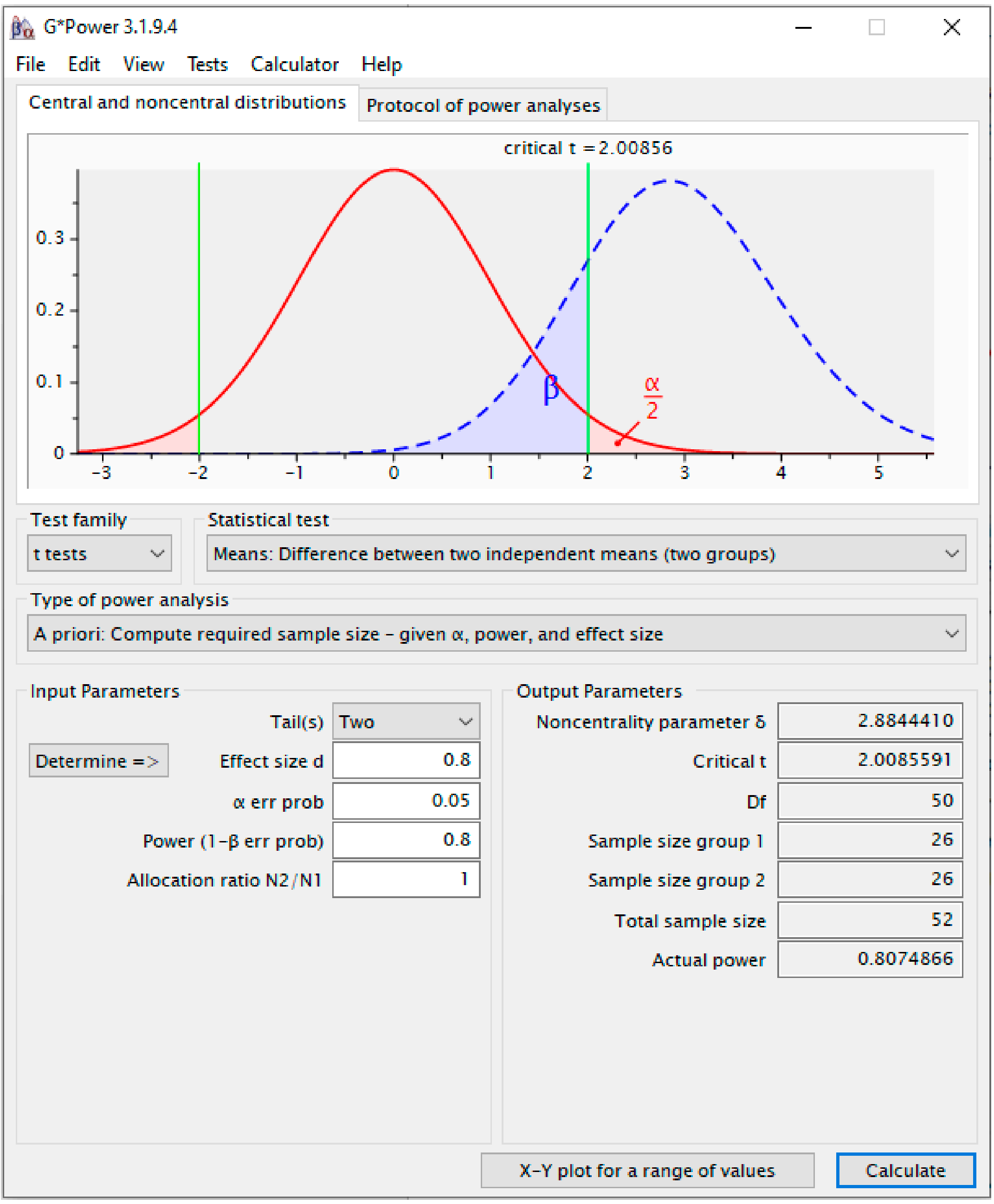

Figure S1. sample size calculation.
